# Genomes from bacteria associated with the canine oral cavity: A test case for automated genome-based taxonomic assignment

**DOI:** 10.1371/journal.pone.0214354

**Published:** 2019-06-10

**Authors:** David A. Coil, Guillaume Jospin, Aaron E. Darling, Corrin Wallis, Ian J. Davis, Stephen Harris, Jonathan A. Eisen, Lucy J. Holcombe, Ciaran O’Flynn

**Affiliations:** 1 Genome Center, University of California, Davis, CA, United States of America; 2 The Ithree Institute, University of Technology Sydney, Ultimo NSW, Australia; 3 The Waltham Centre for Pet Nutrition, Melton Mowbray, Leicestershire, United Kingdom; 4 Evolution and Ecology, Medical Microbiology and Immunology, University of California, Davis, Davis, CA, United States of America; Wilfrid Laurier University, CANADA

## Abstract

Taxonomy for bacterial isolates is commonly assigned via sequence analysis. However, the most common sequence-based approaches (e.g. 16S rRNA gene-based phylogeny or whole genome comparisons) are still labor intensive and subjective to varying degrees. Here we present a set of 33 bacterial genomes, isolated from the canine oral cavity. Taxonomy of these isolates was first assigned by PCR amplification of the 16S rRNA gene, Sanger sequencing, and taxonomy assignment using BLAST. After genome sequencing, taxonomy was revisited through a manual process using a combination of average nucleotide identity (ANI), concatenated marker gene phylogenies, and 16S rRNA gene phylogenies. This taxonomy was then compared to the automated taxonomic assignment given by the recently proposed Genome Taxonomy Database (GTDB). We found the results of all three methods to be similar (25 out of the 33 had matching genera), but the GTDB approach required fewer subjective decisions, and required far less labor. The primary differences in the non-identical taxonomic assignments involved cases where GTDB has proposed taxonomic revisions.

## Introduction

With the ever-decreasing costs of DNA sequencing, it has become far easier and cheaper to sequence bacterial genomes than to analyze them. Understanding the gene content and metabolic pathways of a newly sequenced isolate is a time-consuming and knowledge-intensive process. Another, perhaps underappreciated, bottleneck is properly assigning taxonomy to a genome. This is most often seen with metagenome-assembled genomes (MAGs) which are unidentified prior to sequencing, but is even a problem with cultured isolates. Traditional morphological taxonomic assignment of bacterial isolates is tedious, and the more common approach of 16S rRNA gene PCR followed by Sanger sequencing is often uninformative beyond the genus level. Given the costs, some laboratories sequence isolate genomes directly, with no attempt at prior identification. While some have argued against the need for taxonomic assignment for many aspects of microbial genome analysis (e.g., [1][2]), there are many situations where taxonomy is considered valuable for making use of genomic information (e.g., see [3] and [4]).

There have been a number of proposed attempts to move to a genome-based taxonomy for bacteria and archaea, instead of relying on traditional chemotaxonomic/morphological characteristics of isolates [5][6][7]. These include the use of average nucleotide identity (ANI) [8][9][10][11], concatenated marker gene phylogenies (e.g. SILVA (unpublished) and GTDB [12], and shared protein content [13]. Most of these approaches however rely on a provisional identification (at least to genus), followed by locating and downloading the genomes of close relatives for comparison.

In this work, we briefly describe the genome sequences of 33 bacterial isolates from the canine oral cavity. These isolates were collected as part of a larger project on canine oral health and had a preliminary taxonomy assigned through Sanger sequencing and a sequence identity comparison of the 16S rRNA gene [14,15]. After genome sequencing, we first assigned taxonomy to these isolates based on a manual examination of “whole genome” concatenated marker phylogenetic trees, average nucleotide identity (ANI), and 16S rRNA gene phylogenetic trees. We then compared this taxonomy to automated taxonomic assignments given by the recently proposed Genome Taxonomy Database (GTDB).

## Genomes and taxonomy

### Genome selection

The study design, bacterial isolation, DNA extraction, isolation identification and genome sequencing/assembly have been previously described in our work on the *Porphyromonas* genus [16]. Briefly, bacterial isolates from the canine oral cavity were grown on supplemented Columbia Blood Agar containing 5% defibrinated horse blood (CBA; Oxoid, UK) with or without the addition of 5 mg/L hemin (catalog no. H9039; Sigma) and 0.5 mg/L menadione (catalog no. M5625; Sigma) or Heart Infusion Agar containing 5% defibrinated horse blood (HIA:Oxoid,UK) (Table 1). Aerobes were incubated at 38°C under normal atmospheric conditions for 1–5 days. Microaerophilic and anaerobic strains were incubated at 38°C for 1–21 days in a MACS1000 anaerobic workstation (Don Whitley, UK) with gas levels at 5% oxygen, 10% carbon dioxide, and 85% nitrogen for microaerophiles, and 10% hydrogen, 10% carbon dioxide, and 80% nitrogen for anaerobes. Following DNA extraction, library preparation, and Illumina sequencing, the reads were assembled using the A5-miseq assembly pipeline [17].

**Table 1:** Comparative taxonomy of 33 bacterial strains by three different methods.

Isolate Number	Media	Aerobe/anaerobe	16S taxonomy (Sanger, BLAST)	Manual Taxonomy (S, A, or W)	GTDB Taxonomy	Comments	Accession
ATCC 29435	CBA H&M	Aerobe	Conchiformibius steedae_COT-280	Conchiformibius steedae (S)	Conchiformibius steedae	All species	RQYC00000000
OH1139	CBA	Microaerophile	Staphylococcus epidermidis	Staphylococcus epidermidis (A)	Staphylococcus epidermidis	All species	RQYG00000000
OH1877	CBA	Aerobe	Actinomyces hordeovulneris COT-415	Actinomyces hordeovulneris (A)	Actinomyces_D hordeovulneris	All species	RQYO00000000
OH2158	CBA H&M	Anaerobe	Campylobacter rectus	Campylobacter rectus (A)	Campylobacter_A rectus	All species	RQYP00000000
OH1206	HIA	Anaerobe	Bacillus licheniformis (100%)	Bacillus licheniformis (A)	Bacillus licheniformis	All species	RQYJ00000000
OH3297	CBA	Aerobe	Actinomyces hordeovulneris COT-415	Actinomyces hordeovulneris (A)	Actinomyces_D hordeovulneris	All species	RQYV00000000
OH4621	CBA	Aerobe	Streptococcus minor COT-116	Streptococcus minor (A)	Streptococcus minor	All species	RQZA00000000
OH953	CBA	Aerobe	Streptococcus sanguinis	Streptococcus sanguinis (A)	Streptococcus sanguinis	All species	RQZI00000000
OH1186	CBA H&M	Anaerobe	Desulfovibrio sp. COT-070	Desulfovibrio (S)	Desulfovibrio	All genus	RQYH00000000
OH1287	HIA	Aerobe	Leucobacter sp. COT-429	Leucobacter (S)	Leucobacter	All genus	RQYM00000000
OH2974	CBA	Aerobe	Leucobacter sp. COT-288	Leucobacter (S)	Leucobacter	All genus	RQYU00000000
OH3620	HIA	Anaerobe	Leptotrichia_COT-345	Leptotrichia (S)	Leptotrichia	All genus	RQYW00000000
OH937	HIA	Anaerobe	Prevotella sp. COT-195	Prevotella (S)	Prevotella	All genus	RQZH00000000
OH1220	CBA H&M	Microaerophile	Fretibacterium sp. COT-178	Fretibacterium (S)	Fretibacterium	All genus	RQYL00000000
OH1205	HIA	Anaerobe	Alloprevotella sp. COT-284	Alloprevotella (S)	F0040 [Alloprevotella]*	All genus	RQYI00000000
OH2545	CBA	Aerobe	Ottowia sp. COT-014	Ottowia (W)	Ottowia	All genus	RQYR00000000
OH741	CBA H&M	Microaerophile	Erysipelotrichaceae [G-1] sp. COT-311	Erysipelotrichaceae (S)	Erysipelotrichaceae	All family	RQZE00000000
OH1209	CBA	Aerobe	Streptococcus sp. COT-279	Desulfovibrio (S)	Desulfovibrio	Mis-identification	RQYK00000000
OH4464	CBA H&M	Anaerobe	Proprionibacterium sp. COT-324	Tesseracoccus (S)	Tessaracoccus	Related genus	RQYZ00000000
OH4692	CBA	Aerobe	Streptococcus pneumoniae COT-348	Streptococcus (S)	Streptococcus	16S species, manual & GTDB genus	RQZB00000000
OH2310	CBA	Aerobe	Xenophilus sp. COT-174	Lampropedia (W)	Lampropedia	Related genus	RQYQ00000000
OH3737	CBA	Aerobe	Xenophilus sp. COT-264	Lampropedia (W)	Lampropedia	Related genus	RQYX00000000
OH1047	CBA H&M	Anaerobe	Bacteroides heparinolyticus COT-310	Prevotella heparinolyticus (S)	Bacteroides	Official nomenclature change	RQYF00000000
OH1426	CBA H&M	Anaerobe	Tannerella forsythus COT-023	Tannerella forsythia (S)	Tannerella	GTDB genus, manual & 16S species	RQYN00000000
OH2617	CBA H&M	Anaerobe	Tannerella forsythus COT-023	Tannerella forsythia (S)	Tannerella	GTDB genus, manual & 16S species	RQYS00000000
OH4460	CBA H&M	Anaerobe	Fusobacterium canifelinum COT-188	Fusobacerium canifelinum (S)	Fusobacterium	GTDB genus, manual & 16S species	RQYY00000000
OH5050	CBA	Aerobe	Actinomyces bowdenii COT-413	Actinomyces bowdenni (S)	Actinomyces	GTDB genus, manual & 16S species	RQZC00000000
OH770	CBA	Aerobe	Actinomyces canis COT-409	Actinomyces canis (S)	Actinomyces_B	GTDB genus, manual & 16S species	RQZF00000000
OH5060	CBA H&M	Anaerobe	Fusobacterium sp. COT-236	Fusobacterium nucleatum (W)	Fusobacterium	GTDB & 16S genus, manual species	RQZD00000000
OH887	CBA H&M	Anaerobe	Proprionibacterium sp. COT-365	Propionibacterium propionicum (S)	Pseudopropionibacterium	GTDB proposed taxonomic change	RQZG00000000
6824 SB	HIA	Anaerobe	Lachnospiraceae XIVa [G-6] sp. COT-073	Clostridiales (S)	Lachnospirales	GTDB proposed taxonomic change	RQYD00000000
OH1046	CBA H&M	Anaerobe	Atopobium parvulum	Coriobacterineae (W)	Atopobiaceae	GTDB proposed taxonomic change	RQYE00000000
OH2822	CBA H&M	Anaerobe	Proprionibacterium sp. COT-296	Propionibacterium propionicum (S)	Pseudopropionibacterium	GTDB proposed taxonomic change	RQYT00000000

CBA: Columbia Blood Agar, HIA: Heart Infusion Agar, H&M: Hemin and Menadione.

*"F0400" is actually a strain name, used as a placeholder by GTDB when the authors believe that genome belongs in a new genus, for which no type representative is present. (S) is for strains in the manual approach whose taxonomy was assigned using 16S rRNA gene trees, (A) for those for which ANI was used, and (W) for those for which a WGS tree was used. The comments column describes the way in which each of the three methods differs or is the same.

The remaining non-*Porphyromonas* genomes were further screened by a combination of assembly metrics and CheckM [18] to estimate completeness and contamination. We chose for further study those that appeared to be the highest quality using admittedly somewhat arbitrary cutoffs of (a) fewer than 350 contigs in the assembly, (b) CheckM contamination score of <3%, and (c) CheckM completeness score of >90%. A subset of 33 genomes meeting these criteria was chosen to study in more detail.

### Preliminary taxonomic identification

All isolates were given a preliminary identification based on analysis of 16S rRNA gene sequences generated via Sanger sequencing (F24/Y36(9-29F/1525-1541R) primers). The 16S rRNA gene sequences were searched using BLAST [19] against an in-house canine oral microbiome database. This database contained 460 published 16S rRNA gene sequences obtained from canine oral taxa (Genbank accession numbers JN713151-JN713566 & KF030193-KF030235 [15]. In addition, sequences were searched against the RDP 16S rRNA database v10_31 [20]. Taxonomic identifications were made using percent identity cutoffs of 98.5% (for species), 94% (for genus), and 92% (for family).

### Traditional taxonomic identification

The contigs comprising the genomes were first uploaded to the RAST server [21] for annotation. The results archives were downloaded using the RAST API and then searched for full-length 16S rRNA gene sequences by scanning the annotations using the “SSU rRNA” tag and filtering for length greater than 1 kb. These sequences were uploaded to the Ribosomal Database Project (RDP) [20] and incorporated into their alignment. For first-pass taxonomic identification, an alignment and phylogenetic tree was generated using SSU-ALIGN [22] and FastTree 2 [23] of all ~12,000 type strain 16S rRNA gene sequences from RDP, along with our 33 sequences from RAST. This provided us with the general region of the tree for each isolate or group of isolates.

Next, we generated a series of concatenated “whole genome sequence” (WGS) marker trees for each isolate or group of isolates as described below. All available genome sequences from the genus or family of interest were downloaded from GenBank, except in cases where more than 500 genomes were available. In those genome-rich genera (*Bacillus*, *Clostridium*, *Streptococcus*, *Staphylococcus*, *Campylobacter*), the WGS trees were built with only a subset of genomes, selected from the type strain results. For example, *Staphylococcus* contains ~40 species, but only genomes from those species closest (by eye in the tree) to our isolate of interest were needed to build a useful WGS tree.

For all WGS trees, an outgroup genome(s) was chosen from nearby in the NCBI taxonomy (e.g. another genus in the same family). The file names and sequences were reformatted for easier visualization. The assemblies were then screened for 37 core maker genes [24] using PhyloSift [25] in the search and align mode using “isolate” and “besthit” flags. PhyloSift concatenates and aligns the hits of interest then the sequences are subsequently extracted from the PhyloSift output files and added to a single file for tree-building. An approximately maximum-likelihood tree was then inferred using FastTree2 with default parameters [23].

For all isolates where the WGS tree indicated a possible placement into a well-defined clade with sequenced genomes, we downloaded the type strain genome sequences for every member of that genus/family from Refseq at NCBI. These were used to create an ANI (average nucleotide identity) matrix using FastANI [26] for each group and anything having an ANI >95% to a type strain was considered to belong to that species [8][27].

For the majority of taxa, the WGS tree and ANI matrix were still inadequate to assign species level taxonomy, due to a paucity of genome sequences for many groups. In those cases, we relied solely on the 16S rRNA gene for taxonomy, but using phylogeny instead of just sequence identity as in the first pass. This was again accomplished through RDP, by downloading all sequences for a given genus/family, inferring a phylogenetic tree, and looking for well-supported placement into a monophyletic clade. For all groups with fewer than 3000 sequences, the alignment was downloaded directly from RDP, for larger groups the sequences were downloaded and the alignment generated with SSU-ALIGN. All alignments were cleaned to remove problematic characters in the headers using a custom script [28] and all trees were inferred using FastTree2 with default parameters.

Final taxonomic assignments were based on a taxa-dependent combination of the WGS trees, the ANI results, and the 16S rRNA gene trees (Table 1). First priority was given to the ANI results, then to placement within a well-supported WGS tree with monophyletic clades, and then finally to 16S rRNA gene-based results. As a result of inadequate mapping between phylogeny and taxonomy, some isolates were only assigned to the genus or family level, and in one case the order level. Examples of both informative and non-informative WGS/16S trees, along with a sample ANI matrix, can be found in the Supplemental Materials. Of the remaining WGS/16S trees and ANI results, those that were informative in determining taxonomy can be found at (doi: 10.25338/B8801B).

### Genome taxonomy database

The Genome Taxonomy Database (GTDB) is a recently proposed system attempting to create a genomic-based, standardized bacterial taxonomy [12]. The system uses 120 single-copy conserved marker genes to generate a phylogenetic tree of 94,759 genomes, and analysis of the topology of this tree was then used to propose large-scale revisions to bacterial taxonomy. During our work on this project, a tool based on GTDB was release; “GTDB-Tk” [29] attempts to automate taxonomic assignment for genomes, based on their revised GTDB taxonomy. The tool uses a combination of phylogenetic tree topology, ANI values, and relative evolutionary divergence (RED). We screened our 33 isolates using this tool and compared the taxonomic assignments to the manual process above (Table 1). Note that an alphabetic suffix in the GTDB taxonomy (e.g. “*Actinomyces_B*”) indicates that, within the GTDB tree, the genus does not belong to a monophyletic group with the type species of that genus.

## Discussion

Here we present the genomes of 33 bacterial isolates from the canine oral cavity. Some are from groups for which some of the members of these groups are known to be involved in human and canine oral health (e.g. *Actinomyces* and *Fusobacterium)* and others have not been previously suggested to play such a role. A few of our isolates appear to be phylogenetically novel and potentially of interest for further study. For example Canine Oral Taxon number 073 (COT073) and bacterial isolate number OH741 were only identified to the order and family level respectively.

The difference in labor required by the two genomic methods of taxonomic assignment was noticeable, with the manual method having taken two weeks of daily downloads, alignments, ANI comparisons and tree-building whereas the GTDB automated method ran overnight. The latter also is much less subjective (for the user) than the former. A comparison of the three approaches to taxonomic assignments shows a very high degree of similarity (Table 1).

For 51% (17/33) of isolates the three methods gave identical results; for 18.2% (6/33) of isolates the 16S and manual methods gave the same results; and in one case the 16S and GTDB methods gave the same results. Finally, for 12% (4/33) of isolates all three methods gave different results to each other i.e. different genera within same family or different levels of assignment (granularity/resolution) within the same branch of the tree. Most of the differences between the GTDB taxonomy and the other approaches appears to be due to proposed taxonomic revisions by the GTDB group. For example COT073 was placed within the Clostridiales order in the manual taxonomy, but that order was subdivided into several new groups in the GTDB taxonomy, based on calculations of relative evolutionary divergence (RED) within that group.

Our results suggest that the use of the automated GTDB tool to assign taxonomy to unidentified bacterial isolates is less subjective and much faster than manual assignment, while giving very similar results (e.g. identical taxonomy, difference only in taxonomic rank, or proposed taxonomic changes). Both genomic methods offer improved taxonomic resolution relative to analysis of just 16S rRNA gene sequences, at the additional cost/burden of requiring the entire genome. Finally, we expect these genomes will be of use to researchers studying canine oral health since the vast majority of closely related isolates so far sequenced have been collected from humans.

## Accession numbers

The whole-genome shotgun projects have been deposited at DDBJ/ENA/GenBank under the accession numbers RQYC00000000-RQZI00000000. The versions described in this paper are versions RQYC01000000-RQZI01000000. These strains were submitted to NCBI using our “manual taxonomy” assignments, since the GTDB taxonomy is not yet widely accepted. However, for some of the isolates, NCBI made their own minor taxonomic revisions.

## Supporting information

S1 Fig A taxonomically inconclusive WGS tree for isolate OH770. The isolate is not found within a clade of other sequenced isolates with species-level taxonomy.
(PDF)

S2 Fig A taxonomically inconclusive 16S rRNA gene tree for isolate OH1287. Taxonomy is not congruent with phylogeny and many neighboring sequences are only identified to the genus level.
(PDF)

S3 Fig A taxonomically informative 16S rRNA gene tree for isolate OH5050. The isolate is found in a monophyletic clade and the name given to the closest relatives is not found elsewhere in the tree.
(PDF)

S1 Table A sample ANI result for isolate OH3297. The isolate is ~99% identical to both OH1877 and to *Actinomyces hordeovulneris*. The ANI drops off quite rapidly to other members of the genus.
(DOCX)
