# Supplementary figures and images for: Genomes from bacteria associated with the canine oral cavity: A test case for automated genome-based taxonomic assignment
Source: PLoS One. 2019 Jun 10;14(6):e0214354. doi: 10.1371/journal.pone.0214354 (PMC6557473; doi:10.1371/journal.pone.0214354)

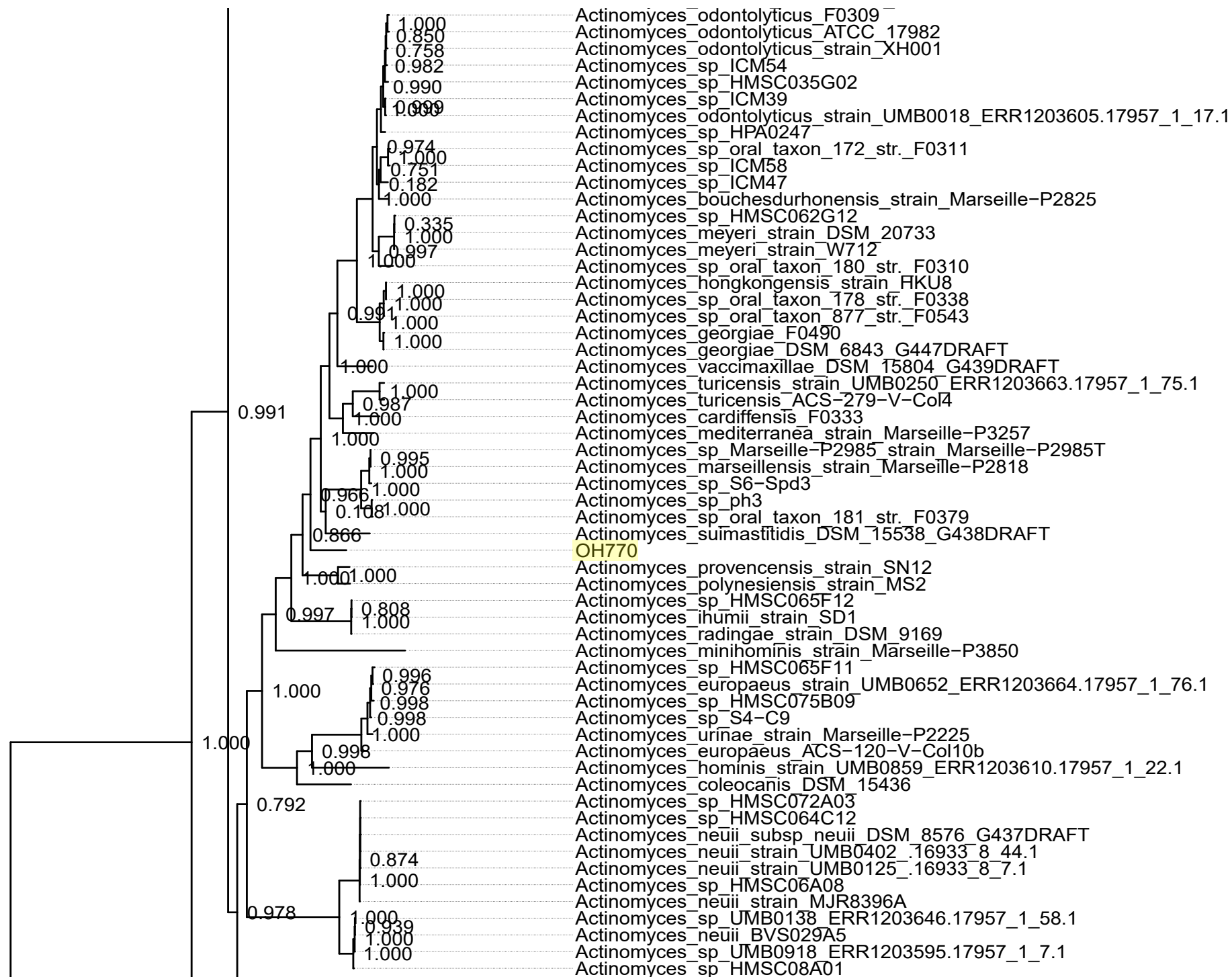

Supplement: S1 Fig — The isolate is not found within a clade of other sequenced isolates with species-level taxonomy. (PDF) [file pone.0214354.s001.PDF]

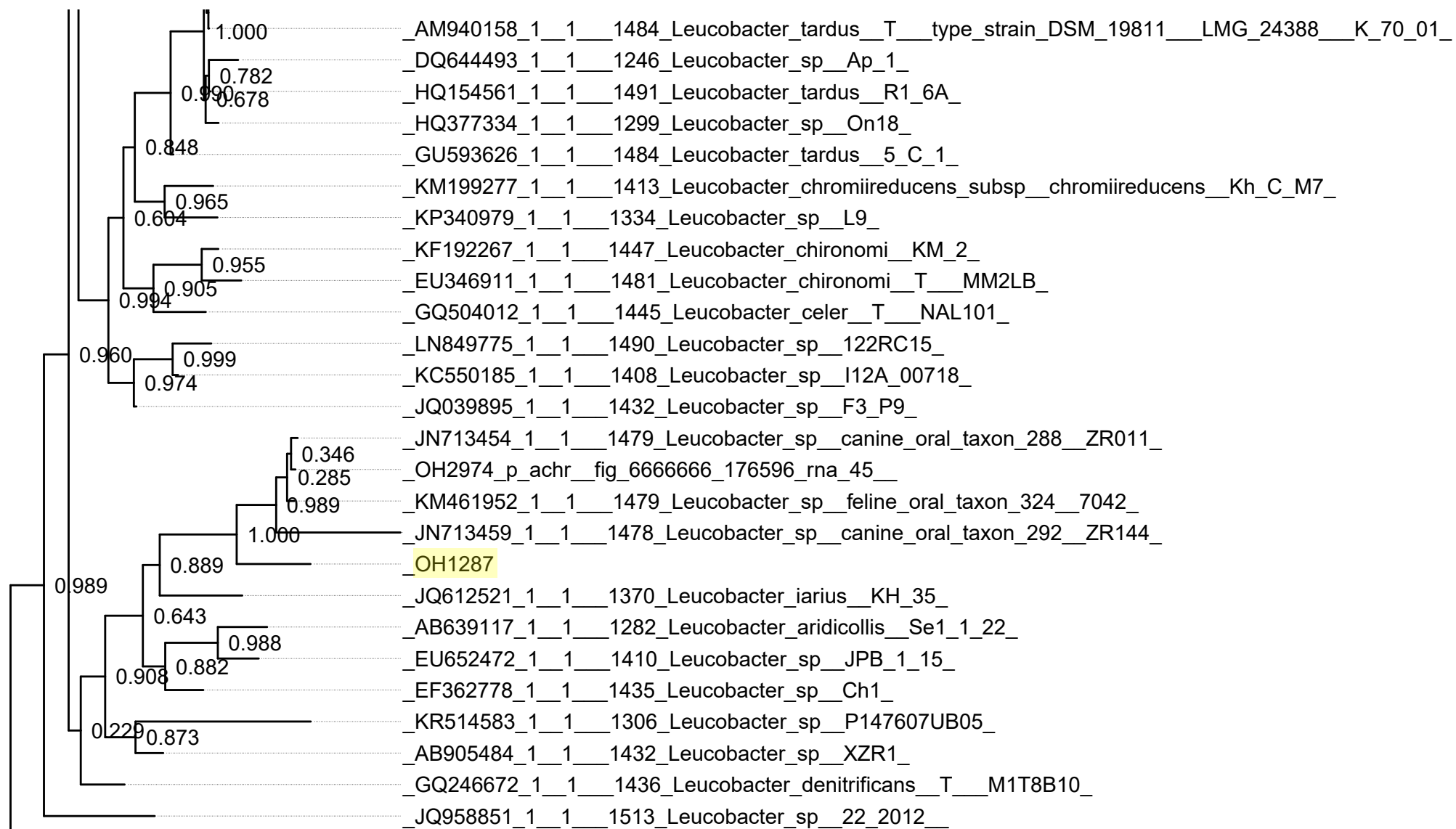

0.0

0.1

0.2

0.3

0.4

0.5

Supplement: S2 Fig — Taxonomy is not congruent with phylogeny and many neighboring sequences are only identified to the genus level. (PDF) [file pone.0214354.s002.PDF]

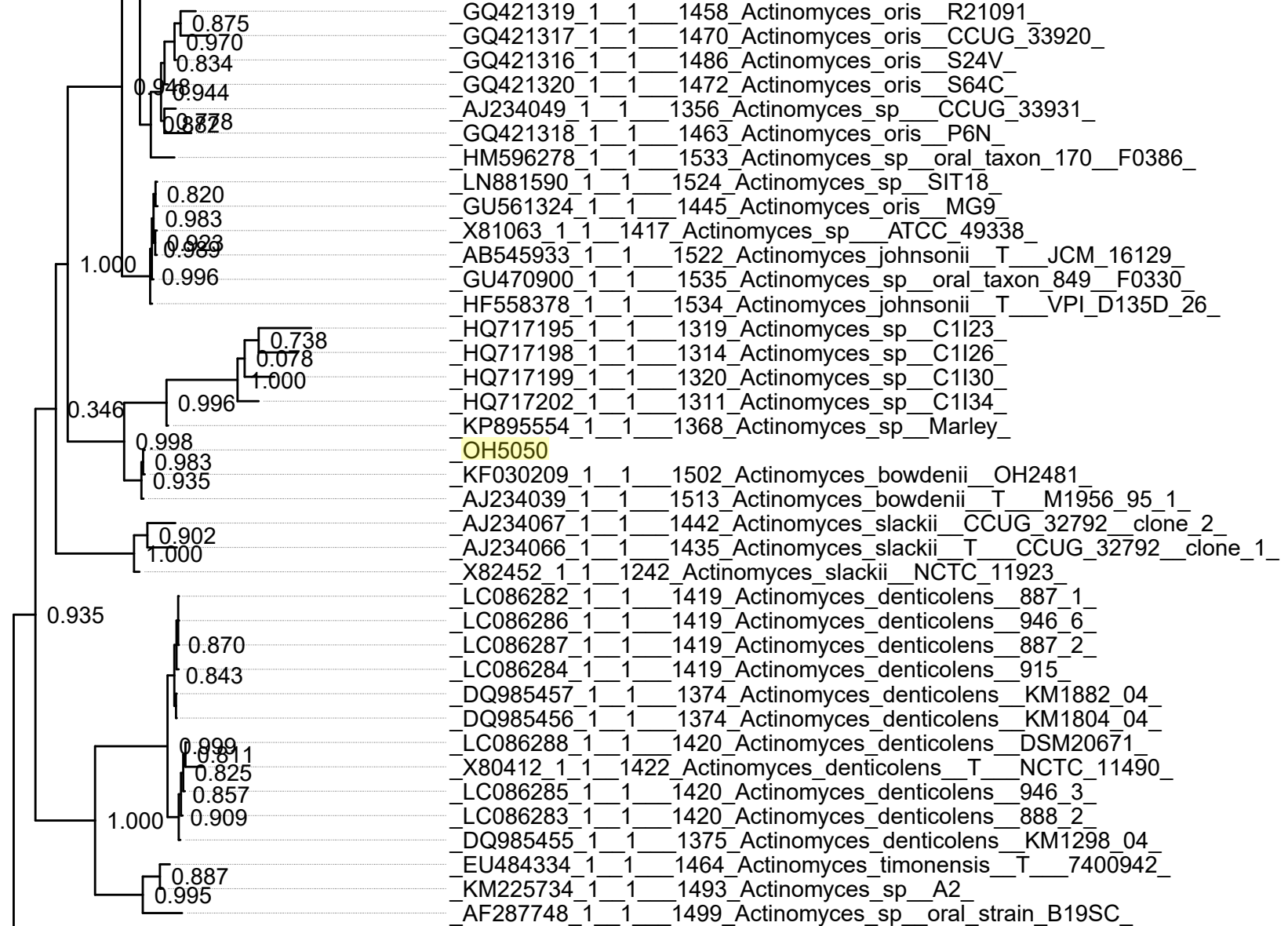

0.0

0.2

0.4

Supplement: S3 Fig — The isolate is found in a monophyletic clade and the name given to the closest relatives is not found elsewhere in the tree. (PDF) [file pone.0214354.s003.PDF]
