# Supplementary material for: Genomes from bacteria associated with the canine oral cavity: A test case for automated genome-based taxonomic assignment
Source: PLoS One. 2019 Jun 10;14(6):e0214354. doi: 10.1371/journal.pone.0214354 (PMC6557473; doi:10.1371/journal.pone.0214354)
Supplement: S1 Table — The isolate is ~99% identical to both OH1877 and to Actinomyces hordeovulneris. The ANI drops off quite rapidly to other members of the genus. (DOCX) [file pone.0214354.s004.docx]

**Supplemental Table 1**

| **Query** | **Subject** | **ANI** |
| --- | --- | --- |
| OH3297 | OH3297 | 100 |
| OH3297 | OH1877 | 98.9637 |
| OH3297 | Actinomyces_hordeovulneris_strain_DSM_20732 | 98.9036 |
| OH3297 | Actinomyces_meyeri_strain_DSM_20733 | 77.6951 |
| OH3297 | Actinomyces_slackii_ATCC_49928_G316DRAFT | 77.6356 |
| OH3297 | Actinomyces_timonensis_DSM_23838_strain_7400942 | 77.6052 |
| OH3297 | Actinomyces_mediterranea_strain_Marseille-P3257 | 77.5283 |
| OH3297 | OH5050 | 77.4716 |
| OH3297 | Actinomyces_urogenitalis_DSM_15434 | 77.389 |
| OH3297 | Actinomyces_naeslundii_strain_NCTC_10301_NCTC10301 | 77.3848 |
| OH3297 | Actinomyces_massiliensis_4401292 | 77.3507 |
| OH3297 | Actinomyces_denticolens_strain_DSM_20671 | 77.3352 |
| OH3297 | Actinomyces_bouchesdurhonensis_strain_Marseille-P2825 | 77.2802 |
| OH3297 | Actinomyces_odontolyticus_ATCC_17982 | 77.1878 |
| OH3297 | Actinomyces_gaoshouyii_strain_pika_113 | 77.1605 |
| OH3297 | Actinomyces_dentalis_DSM_19115_G446DRAFT | 77.0652 |
| OH3297 | Actinomyces_provencensis_strain_SN12 | 77.0571 |
| OH3297 | Actinomyces_gerencseriae_DSM_6844_G448DRAFT | 77.0556 |
| OH3297 | OH770 | 77.0508 |
| OH3297 | Actinomyces_polynesiensis_strain_MS2 | 77.0011 |
| OH3297 | Actinomyces_hongkongensis_strain_HKU8 | 76.9649 |
| OH3297 | Actinomyces_radicidentis_strain_CCUG_36733 | 76.896 |
| OH3297 | Actinomyces_georgiae_DSM_6843_G447DRAFT | 76.8312 |

A sample ANI result for isolate OH3297. The isolate is ~99% identical to both OH1877 and to *Actinomyces hordeovulneris*. The ANI drops off quite rapidly to other members of the genus.
